# Novel insight into streptozotocin-induced diabetic rats from the protein misfolding perspective

**DOI:** 10.1038/s41598-017-11776-y

**Published:** 2017-09-14

**Authors:** Edgar Leyva-García, Reyna Lara-Martínez, Liborio Morán-Zanabria, Cristina Revilla-Monsalve, Luis Felipe Jiménez-García, Norma Oviedo, Chiharu Murata, Eulalia Garrido-Magaña, Nelly F. Altamirano-Bustamante, Myriam M. Altamirano-Bustamante

**Affiliations:** 1 Unidad de Investigación en Enfermedades Metabólicas, Centro Médico Nacional Siglo XXI, Instituto Mexicano del Seguro Social, Mexico city, Mexico; 2 Facultad de Ciencias – UNAM, Mexico city, Mexico; 3 Unidad de Investigación en Inmunología e Infectología, Centro Médico Nacional La Raza, Instituto Mexicano del Seguro Social, Mexico city, Mexico; 4 Instituto Nacional de Pediatría, Mexico city, Mexico; 5 Servicio de Endocrinología, UMAE Hospital de Pediatría, Centro Médico Nacional Siglo XXI, IMSS, Mexico city, Mexico

## Abstract

Protein folding is a process of self-assembly defined by the sequence of the amino acids of the protein involved. Additionally, proteins tend to unfold, misfold and aggregate due to both intrinsic and extrinsic causes. Human islet amyloid polypeptide (hIAPP) aggregation is an early step in diabetes mellitus. However, the aggregation of rat IAPP (rIAPP) remains an open question. Adult female Sprague-Dawley rats weighing 150–250 g were divided into two groups. The experimental group (streptozotocin [STZ]) (n = 21) received an intraperitoneal injection of a single dose of 40 mg/kg STZ. We used the mouse anti-IAPP antibody and the anti-amyloid oligomer antibody to study the temporal course of rIAPP oligomerization during STZ-induced diabetes using a wide array of methods, strategies and ideas derived from biochemistry, cell biology, and proteomic medicine. Here, we demonstrated the tendency of rIAPP to aggregate and trigger cooperative processes of self-association or hetero-assembly that lead to the formation of amyloid oligomers (trimers and hexamers). Our results are the first to demonstrate the role of rIAPP amyloid oligomers in the development of STZ-induced diabetes in rats. The IAPP amyloid oligomers are biomarkers of the onset and progression of diabetes and could play a role as therapeutic targets.

## Introduction

Protein folding is the touchstone of proteomic medicine: it changes the current physio-pathological, diagnostic and therapeutic paradigm. Although some proteins have an innate tendency to acquire native conformations, alternate folding, misfolding and aggregation coexist in many, if not all, proteins. These phenomena cause the formation of soluble amyloid oligomers, proto-filaments, amyloid fibres and are the physio-pathological basis of conformational diseases (CDs), including diabetes mellitus, Alzheimer’s disease and cancer^1–9^. CDs are catastrophic diseases because of their clinical complexity as well as high economic and social costs^10–12^.

The islet amyloid polypeptide (IAPP) - also known as amylin - is a 37-residue peptide produced in β cells in the pancreas and is synthesized, processed and secreted along with insulin^13–15^. Recent studies have indicated that the aggregation of human IAPP (hIAPP), especially the formation of soluble pre-fibril aggregates (soluble amyloid oligomers), is a diabetogenic factor that causes apoptosis and progressive β cell failure^4, 8, 10, 15–21^, rendering these cells unable to respond to the demand to compensate insulin resistance^20^. β cell membranes adjacent to biosynthetic deposits *in vivo* and *in vitro* are visibly interrupted, disrupting the cycle of membrane proteins. These soluble amyloid oligomers have low molecular weights and exhibit similar structural features, such as β-sheet-rich structures, physicochemical properties and immunogenic reactivities^7–9, 22–26^. They also have stronger cytotoxicity than monomers and fibres, can be recognized by the conformational anti-amyloid oligomer antibody (A11) and can also form fibres^7–9, 22–26^. The formation of IAPP amyloid fibres is a characteristic of diabetes mellitus^13, 15, 27, 28^, and over 90% of humans and animals (i.e., cats and monkeys) with type 2 diabetes mellitus (DM2) have amyloid deposits located in atrophic areas of β cells in the pancreas^13, 27, 29, 30^. However, the aggregation of rat IAPP (rIAPP) remains an open question^31–37^. In this work, we performed a cross-functional study to determine whether rIAPP aggregation is a step in streptozotocin (STZ)-induced diabetes in rats. This model of diabetes is excellent for testing the hypothesis that IAPP oligomers are early biomarkers of the onset and progression of diabetes and could play a role as therapeutic targets.

## Results

### Experimental model of STZ-induced diabetes

When treated with STZ, the animals developed severe hyperglycaemia, which differed substantially from the levels of the control rats. The serum glucose levels of the animals on the day of euthanasia were quantified as the determined capillary glucose levels during the experiment (Supplemental Fig. 1).

The diabetic animals showed no weight gain during the experiment and, at the end, they presented a significant decrease in body weight (Supplemental Fig. 2). This finding is consistent with the sustained increase of glycaemia and various metabolites, such as cholesterol and triglycerides.

### To rIAPP-aggregate or not to rIAPP-aggregate? That is the question in STZ-induced diabetes in rats

**Figure 1:**
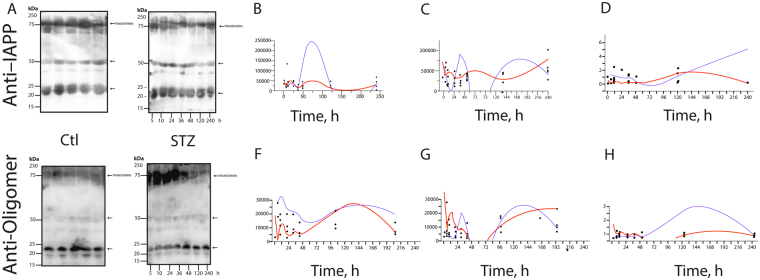
Immunoblot analysis of the anti-IAPP (**A**–**D**) and anti-amyloid oligomers (**E**–**H**) antibodies in the sera of control and STZ-treated animals; (**B**,**C**) present the densitometry results of the trimer and hexamer detected with the anti-IAPP antibody, respectively. Changes in the IAPP-trimers (12 kDa) (**B**) and hexamers (25 kDa) (**C**) in the sera of diabetic rats (blue line) and control rats (red line) throughout the follow-up. The curves were adjusted to the data using the spline function (DM: λ = 1 × 10^5^, R^2^ = 0.11; Control: λ = 3 × 10^6^, R^2^ = 0.14). In addition, (**F** and **G**) present the densitometry results of the trimers and hexamers detected for anti-amyloid oligomers (the blue line corresponds to STZ-treated animals, and the red line corresponds to the controls) The curves for the trimers were adjusted to the data with the spline function (DM: λ = 60, R^2^ = 0.0.08; Control: λ = 300, R^2^ = 0.35). The curves for hexamers were adjusted to the data with the spline function (DM: λ = 1 × 10^5^; R^2^ = 0.09, Control: λ = 1 × 10^5^; R^2^ = 0.20). In (**D** and **H**), the ratios are presented of the densitometry results of the anti-oligomer/anti-IAPP trimers and hexamers, respectively, throughout the follow-up. (blue line: STZ; and red line: control). The curves for the trimers were adjusted to the data with the spline function (DM: λ = 1 × 10^5^, R^2^ = 0.02; Control: λ = 1 × 10^5^, R^2^ = 0.34). The curves for the hexamers were adjusted to the data with the spline function (DM: λ = 1 × 10^5^, R_2_ = 0.09; Control: λ = 1 × 10^5^, R^2^ = 0.20).

**Figure 2:**
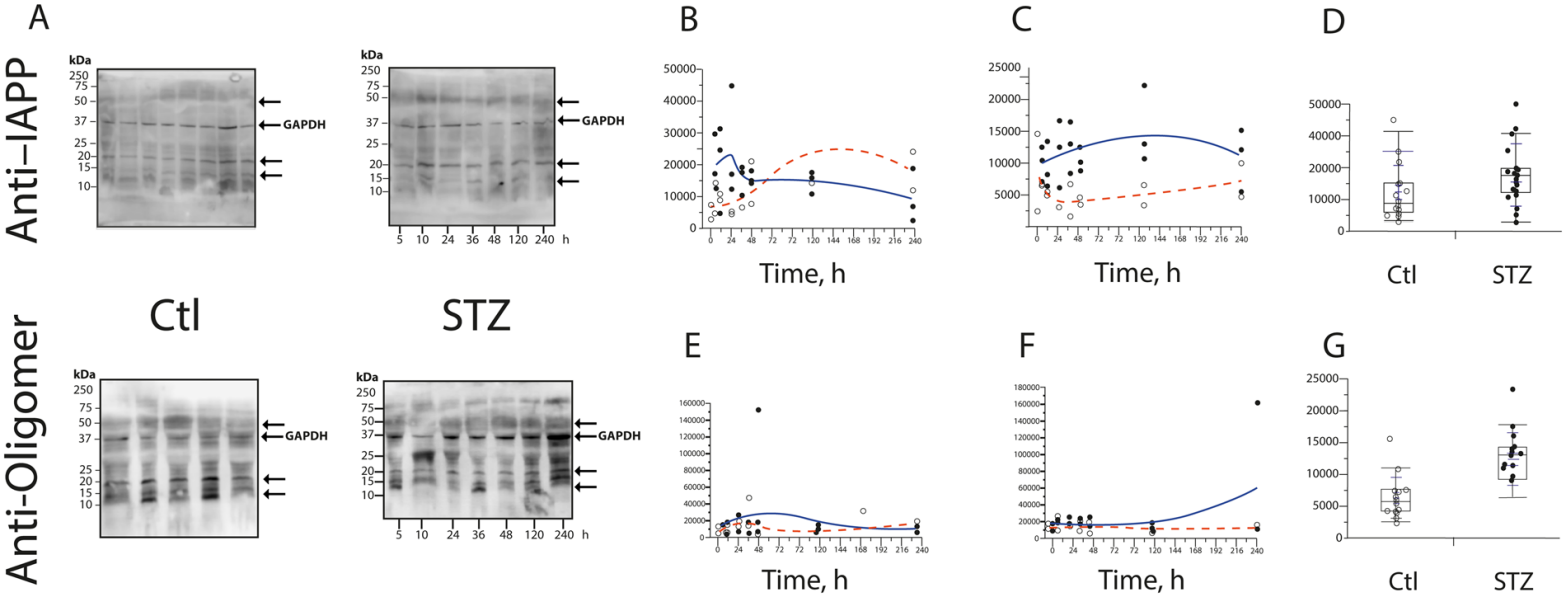
Immunoblot analysis using the anti-IAPP (**B**–**D**) and anti-amyloid oligomers (**E**–**G**) antibodies in pancreas samples. (**B** and **E**) present the densitometry results of the trimers detected with the anti-IAPP and anti-amyloid oligomers antibodies, respectively. In (**C** and **F**), the densitometry results of the hexamers detected with anti-IAPP and anti-amyloid oligomers antibodies, respectively, are presented. The pancreas trimers (**D**) of the control and experimental rats were compared from 0 to 240 treatment hours utilizing the Wilcoxon rank-sum test to assess significant differences (8490 (3,150–34,000) vs. 17,050 (2,645–44,800)) (P = 0.032). The pancreas hexamers (**G**) of the control and experimental rats were compared from 0 to 240 treatments hours utilizing the Wilcoxon rank-sum test to assess significant differences (4,895 (1,640–15,000) vs. 12,380 (5,645–22,850) (P < 0.001).

Although some proteins have an inborn tendency to fold in a native conformation, alternate and anomalous folding as well as aggregation are known to occur in many, if not all, proteins due to the formation of soluble amyloid oligomers and amyloid fibers^1, 5, 21, 38^. Until relatively recently, rIAPP was thought to have a perfectly functional and well-folded structure. We now know that rIAPP can also present disordered elements and tends to misfold and co-aggregate both *in vitro* and *in silico* ^35, 39^. Therefore, we studied the rIAPP aggregation-oligomerization process (10 days) in STZ-induced diabetic rats. First, we compared the sera of STZ-induced animals with the sera of control animals by Western blotting (WB) (Fig. 1A) and the STZ-treated animals’ pancreases with those of the control animals (Fig. 2A). An anti-IAPP antibody or anti-amyloid oligomer antibody was used to identify rIAPP aggregates. The existence of an anti-amyloid oligomer antibody that recognizes all types of amyloid oligomers (independently of the protein sequence) but does not bind to native proteins, monomers or mature amyloid fibres allowed us to detect rIAPP pre-fibrillar oligomers^8, 9, 23, 29^.

Figure 1 shows that in the diabetic animals’ sera, three aggregate species predominate: 12-kDa trimers, 24-kDa hexamers, and 50-kDa dodecamers. Control animals presented significantly different results (Fig. 1(B–D)). Notably, in the WB with the anti-amyloid oligomer antibody, the three aggregate species can be observed (Fig. 1A(F–H)). We also noted that in pancreas samples the same protein bands are present in addition to 55–80-kDa smear bands (Fig. 2). Thus, rIAPP aggregates and forms soluble amyloid oligomers during the development of diabetes (Figs 1 and 2). Based on the published data, Kayed and co-workers demonstrated “that there is an inverse correlation between the size of amyloid protein assemblies and the potency of their exerted toxicity. As the size of the oligomeric assembly increases, its deleterious effects decrease”. Following this reasoning, the oligomers we found are the low-molecular weight ones described by several researchers as cytotoxic. Importantly, these oligomers are recognized by an anti-amyloid oligomer antibody that was determined to recognize pre-fibrillar oligomers that are cytotoxic based on a 3-(4,5-dimethylthiazol-2-yl)-2,5-diphenyltetrazolium bromide (MTT) assay^8, 9, 23, 29^.

This is a remarkable and innovative result because different analyses demonstrate that the aggregation of IAPP in humans and animals (i.e., monkeys and cats), especially the formation of pre-fibrillary aggregates, which are also known as cytotoxic oligomers, is a diabetogenic factor that leads to apoptosis and the progressive failure of β cells^15, 17, 27, 40, 41^. Our results suggest that in the presence of STZ, rIAPP plays a role similar to that of hIAPP. The key point of oligomer formation occurs after 5 hours of diabetes mellitus induction (Figs 1 and 2(B–G)), and on the fifth day, a significant difference was observed relative to the control.

### Ultrastructural immunolocalization of rIAPP amyloid oligomers in the pancreas

**Figure 3:**
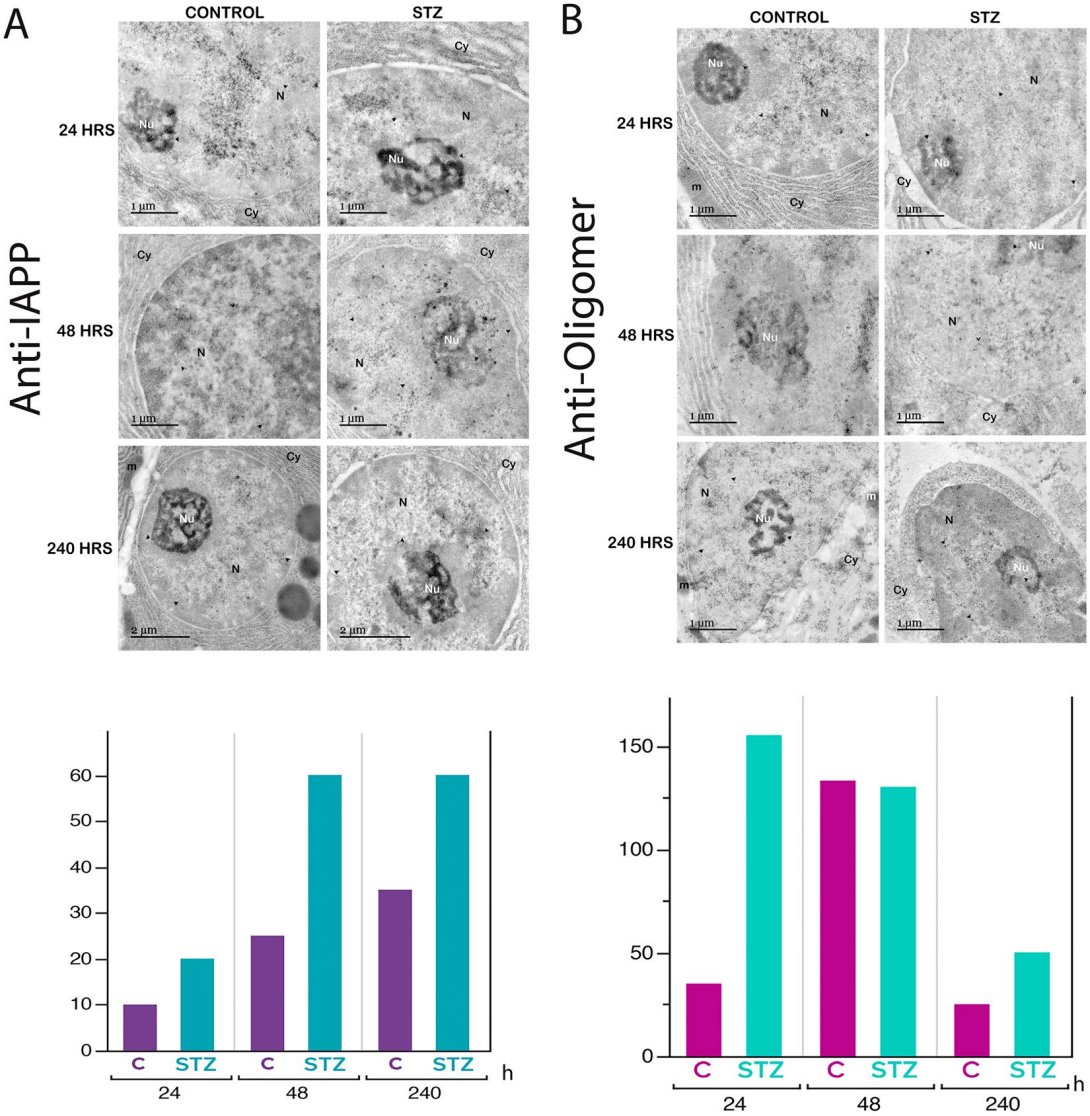
Immunogold labelling in pancreatic cells. (**A**) Amyloid fibril staining with immunogold utilizing the anti-amylin antibody. Numerous gold grains can be seen in the pancreatic cells after 240 hours of STZ treatment; the amount of these grains was decreased in the control cells. (**B**) Pre-fibrillar oligomer staining with immunogold with the anti-amyloid oligomers antibody. The distribution of the gold grains is abundant in the pancreatic cells after 24 hours of treatment. The dark gold grains (arrowhead) reflect the distribution of the antibodies. The labels are principally associated with the nucleolus and nucleoplasm. N, nucleus; nu, nucleolus; cy, cytoplasm; m, mitochondria.

When transmission electron microscopy (TEM) is combined with molecular detection methods, it is a powerful tool for detecting the localization of proteins in intracellular compartments^42^. The EM immunolocalization (EM-I) of rIAPP pre-fibrillar amyloid oligomers in the pancreatic cells of control rats and STZ-treated rats was performed using an anti-IAPP antibody or anti-oligomer antibody followed by secondary antibodies conjugated to gold. The EM-I of both antibodies revealed that the oligomers were mainly distributed in the nucleoplasm of the pancreatic cells and the nucleolus (Fig. 3).

The EM-I of rIAPP revealed a high abundance of rIAPP in the pancreatic cuts of STZ-treated rats, whereas the pancreatic cuts of control rats exhibited low levels of rIAPP (Fig. 3A). Furthermore, the level of rIAPP in the STZ-treated rat increased as the treatment duration increased (Fig. 3A).

The EM-I of amyloid oligomers revealed a high amount of amyloid oligomers in the pancreatic cuts of STZ-treated rats, whereas the pancreatic cuts of control rats exhibited low levels of amyloid oligomers (Fig. 3B). Amyloid oligomers can also be observed in the cytoplasm but only during the 48-hour treatment.

Morphologically, we observed that some aspects of the cell structure were altered, along with the amount of rough endoplasmic reticulum, which diminished in treated rat pancreas cells. We also found dilatation in the nuclear membrane. In the 24-hour control cells, important inter-chromatin granule clusters can be observed, and the level of these clusters decreased as the treatment duration increased. The size and quantity of inter-chromatin granule clusters were reflected in the transcriptional activity, which decreased considerably in the longest-treated cells (Fig. 3).

CDs are characterized by the accumulation of proteins and misfolded peptides in amyloid deposits^5, 6, 38, 43, 44^. This finding is consistent with the results obtained via ultrastructural immunolocalization with the anti-amylin antibody. In the control tissues, small quantities of amyloid rIAPP were observed, but as the treatment duration increased, the amount of amyloid rIAPP increased by 60% (Fig. 3A).

In the tissues from rats treated for shorter periods, the quantity of pre-fibrillar oligomers was elevated. In contrast, in pancreatic cells from rats euthanised after longer STZ treatments, the density of pre-fibrillar oligomers decreased, since this type of oligomer is a transient intermediary species that occurs during fibril formation (Fig. 3B).

### Double immunofluorescence in STZ-treated cells also indicates that the rIAPP was co-localized with amyloid oligomers in the nucleus as well as in the cytoplasm

**Figure 4:**
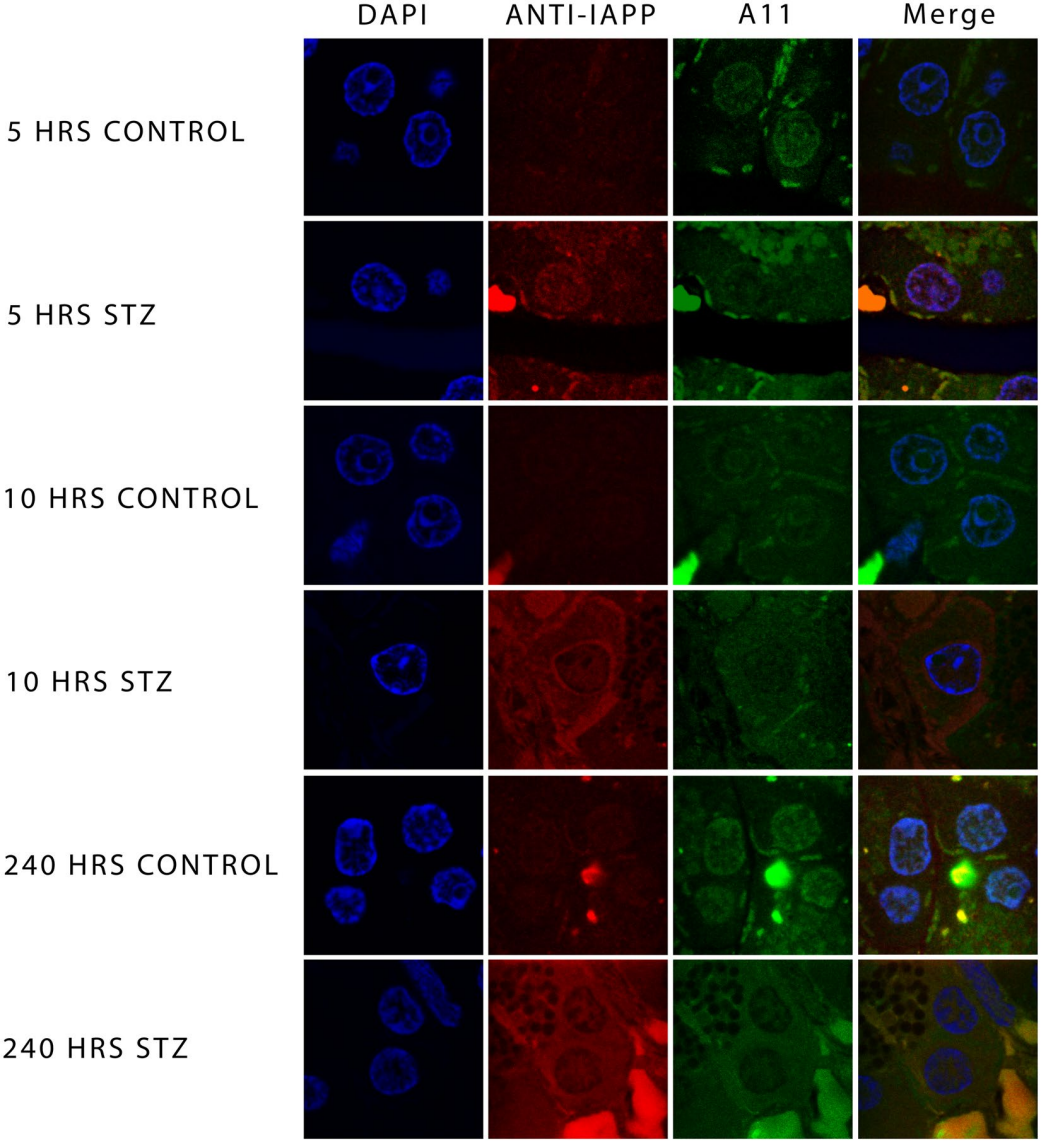
Micrographs of indirect immunofluorescence towards IAPP (red) and amyloid oligomers (green), the cellular nuclei were dyed with DAPI (blue). The figure shows the co-localization of IAPP and amyloid oligomers in the nuclei of β control cells (as shown in the 5-hour merge); however, it can be observed that cells treated with STZ also show the co-localization of both proteins in cytoplasm (STZ 10 and 240-hour merge). IAPP and amyloid oligomers is evidently expressed the most in cells treated with STZ.

Dual labelling indicates that the co-localization of rIAPP and amyloid oligomers corroborated the results presented in the electronic micrographs of ultrastructural immunolocalization (Fig. 3). As shown in Fig. 4, rIAPP detected with the antibodies against IAPP, as well as the prefibrillar oligomers detected with the anti-amyloid oligomers’ antibody, are co-localized in the pancreatic β cells of control slices and those treated with STZ; it is worth pointing out that rIAPP were found to be present in a lesser amount in the control cells than in cells treated with STZ, the same properties can be observed in amyloid oligomers, the expression is greater in cells treated with STZ and lower in control cells; as before, the 240-hour treatment revealed the ultimate expression of this phenomenon.

In contrast, in the ultrastructural immunolocalizations of IAPP and amyloid oligomers uncovered that detection occurs primarily on the nucleus of the cells while using the double immunofluorescence the control cells showed that co-localization of the proteins occurs in the nucleus (blue). While dual staining in STZ-treated cells also indicates that the rIAPP was co-localized with amyloid oligomers in the nucleus as well as the cytoplasm; this is more evident in the 240-hour treatment, where the expression of both proteins is greater (Fig. 4).

**Figure 5:**
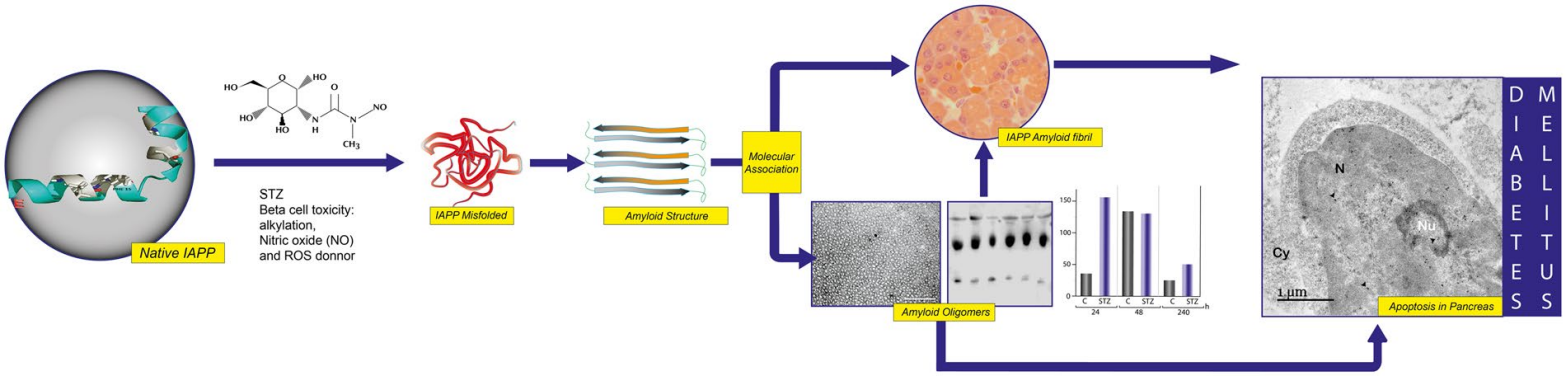
The proposed mechanism of rIAPP aggregation/oligomerization as determined in STZ-induced diabetes in rats. The STZ produced cell stress, increasing protein aggregation and decreasing the capacity for proteostasis. rIAPP undergoes structural perturbations and enters an aggregation-competent state (misfolded), which may even contain β-breakers. The misfolded rIAPP takes on amyloidogenic features, leading to self- and hetero-oligomerization and, finally, conversion into rIAPP fibres. The cytotoxic oligomers cause apoptosis in several cells and the appearance of cross-seeding amyloid structures.

The optical micrographs display pancreatic acini from healthy rats and rats treated with STZ. The acinar cells form groups of grape-like clusters with a basal cytoplasm. When dyed with Congo red, the cytoplasm appears red, and the zymogen granules appear orange (Fig. 5).

## Discussion and Conclusions

### rIAPP-oligomers or Hetero-oligomers?

**Figure 6:**
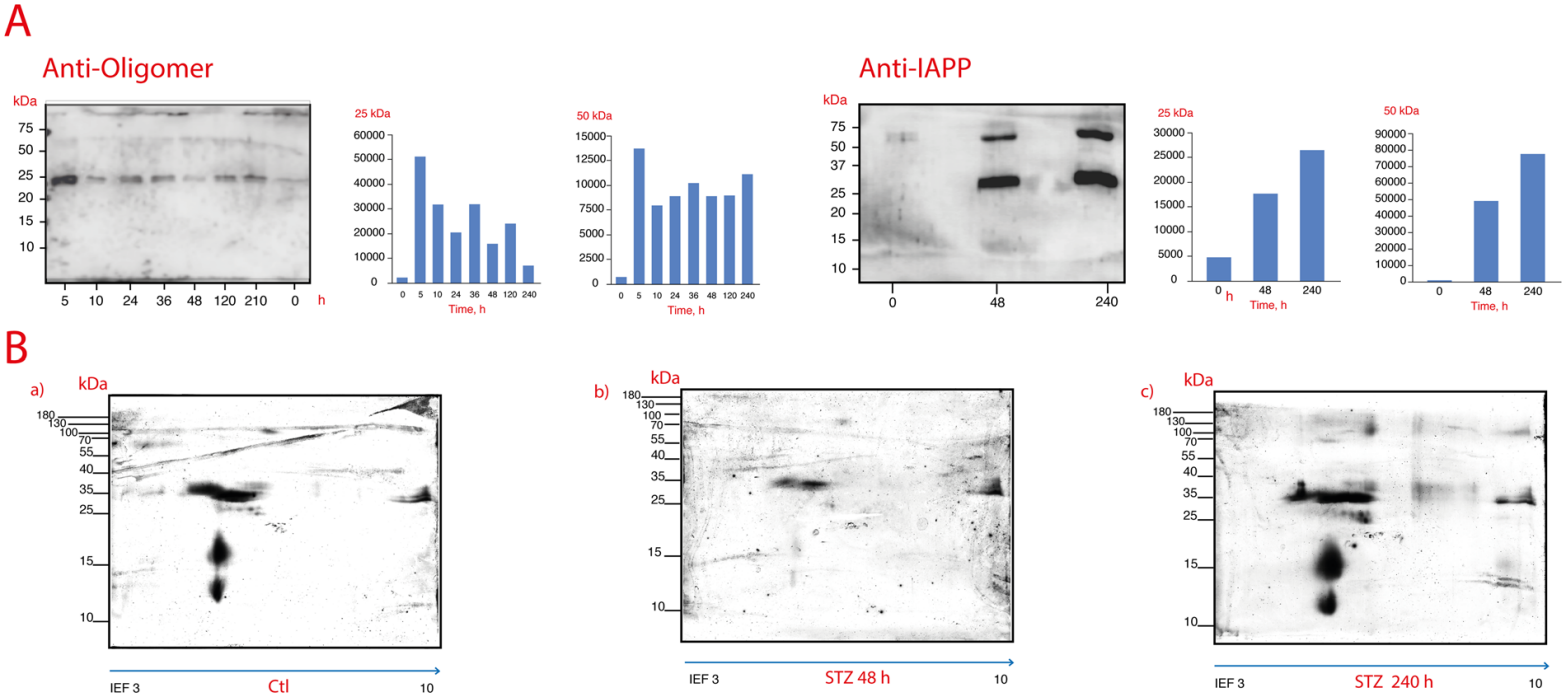
(**A**) Immunoblot analysis using the anti-IAPP and the anti-amyloid oligomers antibody of the serum samples immunoprecipitated with the anti-IAPP antibody from control and STZ-treated animals. (**B**) Representative silver-stained 2D gel of the serum immunoprecipitated with the anti-IAPP antibody; a: control rat; b and c: STZ-induced diabetes for 48 hrs and 240 hrs, respectively.

Immunoprecipitation of the sera from STZ-treated rats over time was achieved with an antibody against IAPP, followed by immunoblotting of the precipitated sample with an anti-amyloid oligomers antibody. This process revealed a main immunoreactive band at 25 kDa, which is faint in the immunoprecipitation results obtained from control rats (Fig. 6A).

Proteins obtained by immunoprecipitation with anti-IAPP from the sera of control and STZ-treated rats were compared by two-dimensional (2D) electrophoresis (Fig. 6B). Several proteins in the sera of STZ-treated rats had high concentrations. The first group corresponds to acidic proteins with high molecular weights (70–80 kDa) and isoelectric points (pIs) of 3.5 to 6.4. The ratio of this group increased from 2 to 24 in the diabetic samples. The second group consists of proteins with molecular weights of 34–45 kDa and pIs of 5.3 to 6. The ratio of this group increased from 2 to 9 in STZ-treated rats. In the profiles of diabetic rats, small basic proteins of 10 and 16 kDa with a pI of 9.2 and basic proteins of 32, 42, 45, 78 and 100 KDa with pIs of 9.6 to 10 could be detected. In contrast, the immunoprecipitation of sera from control rats yielded low protein concentrations.

The anti-amylin-recognized protein bands (10 kDa, 25 kDa and 50 kDa) of the pancreas from STZ-treated rats were selected for proteomic analysis. The analysed protein bands are shown in Table 1. The 10-kDa band was attributed to haemoglobin subunit beta-1, which has a theoretical mass of 13 kDa and a pI of 7.12. The 25-kDa band was attributed to anionic trypsin-1 precursor, which has a molecular weight of 24.7 kDa. The 50-kDa band exhibited score 48.6 to pancreatic alpha amylase, which has a theoretical mass of 57 kDa.

**Table 1:** Assignment of the components of WB-bound proteins from STZ-treated rats by mass spectrometry analysis of in-gel digested protein bands.

PANCREAS	Band [KDa]	Peptides matched	m/z	z	Representative Peptides	Accession UniProt	Protein	% Cov	Theoretical mass [KDa]
	10	4	1126.6666	1	LHVDPENFR	sp|P02091|HBB1_RAT	Hemoglobin subunit beta-1 OS = Rattus norvegicus GN = Hbb PE = 1 SV = 3	48.3	14
			1730.1564	1	LLGNMIVIVLGHHLGK				
			1090.6842	1	VINAFNDGLK				
			1274.845	1	LLVVYPWTQR				
		2	1048.7216	1	TLYGIYYR	tr|F1LNA9|F1LNA9_RAT	Colipase OS = Rattus norvegicus GN = Clps PE = 4 SV = 1	42	9.3
			2069.2456	1	SIIGAITNTNYGVCLDSTR				
	25	2	2211.178	1	LGEHNINVLEGDEQFINAAK	gi|6981420	anionic trypsin-1 precursor [Rattus norvegicus]	16.3	24.7
			2211.1821	1	LGEHNINVLEGDEQFINAAK				
			2211.1838	1	LGEHNINVLEGDEQFINAAK				
			2211.1873	1	LGEHNINVLEGDEQFINAAK				
			2212.1572	1	LGEHNINVLEGDEQFINAAK				
			2212.2019	1	LGEHNINVLEGDEQFINAAK				
			2283.248	1	IIKHPNYSSWTLNNDIMLIK				
			2211.178	1	LGEHNINVLEGDEQFINAAK				
			2211.1821	1	LGEHNINVLEGDEQFINAAK				
		2	1226.5658	1	QEAEEDLPSAR	gi|6981470	lithostathine precursor [Rattus norvegicus]	22.2	18.7
			1226.5802	1	QEAEEDLPSAR				
			1409.6453	1	SWDTGYPNNSNR				
			1409.6556	1	SWDTGYPNNSNR				
	50	10	1271.7212	1	TAIVHLFEWR	gi|672043968	PREDICTED: pancreatic alpha-amylase [Rattus norvegicus]	48.6	57
			1427.7424	1	ALVFVDNHDNQR				
			1520.7184	1	NWGEGWGFVPTDR				
			1615.826	1	GHGAGGASILTFWDAR				
			1690.9622	1	LSGLLDLALDKDYVR				
			1697.89	1	MAVGFMLAHPYGFTR				
			2257.1772	1	AHFSISNSAEDPFIAIHADSK				
			2332.0625	1	EVTINPDTTCGNDWVCEHR				
			2607.2803	1	NVVNGQPFANWWDNGSNQVAFSR				
			1890.9768	1	VADYMNHLIDIGVAGFR				
	50	7	1514.9233	1	EFDSDMDVGDLQK	sp|P27657|LIPP_RAT	Pancreatic triacylglycerol lipase OS = Rattus norvegicus GN = Pnlip PE = 1 SV = 1	48.2	33
			1700.1111	1	VTGHILVSLFGNGGNSK				
			1719.1016	1	FIWYNNVINPTLPK				
			1790.045	1	FLLYTNENQDNYQK				
			1929.2241	1	NILSQIVDIDGIWEGTR				
			2093.2769	1	ITGLDAAEPYFQGTPEEVR				

Our results suggest that rIAPP forms homo-oligomers, as the 2D gel contains protein bands with several molecular weights (12–100 kDa) and a pI of 10, which corresponds to the pI of IAPP (Fig. 6B). This result confirms the rIAPP homo-oligomerization observed *in silico* ^35^.

rIAPP may also hetero-oligomerize with other proteins. Indeed, we found protein bands with different pIs and molecular weights in the 2D gels (Fig. 6B). Furthermore, some proteins could be candidates for co-aggregation with rIAPP, as suggested by the proteomic analysis (Table 1). Wiltzius and co-workers confirmed the heterodimerization of IAPP with insulin^39^, and the binding to catalase^45^. Further studies are required to unveil the molecular network of rIAPP molecular co-aggregation and the role of these aggregates in diabetes mellitus as a CD.

### Assessment of hexamers as an early diagnostic test and biomarker for diabetes

The ratio of the densitometry results of the trimers detected by the anti-oligomer antibody to those of the trimers detected by anti-IAPP (Fig. 1(D)) or the corresponding hexamers (Fig. 1(H)) reveals the differences between the control and diabetic animals.

**Figure 7:**
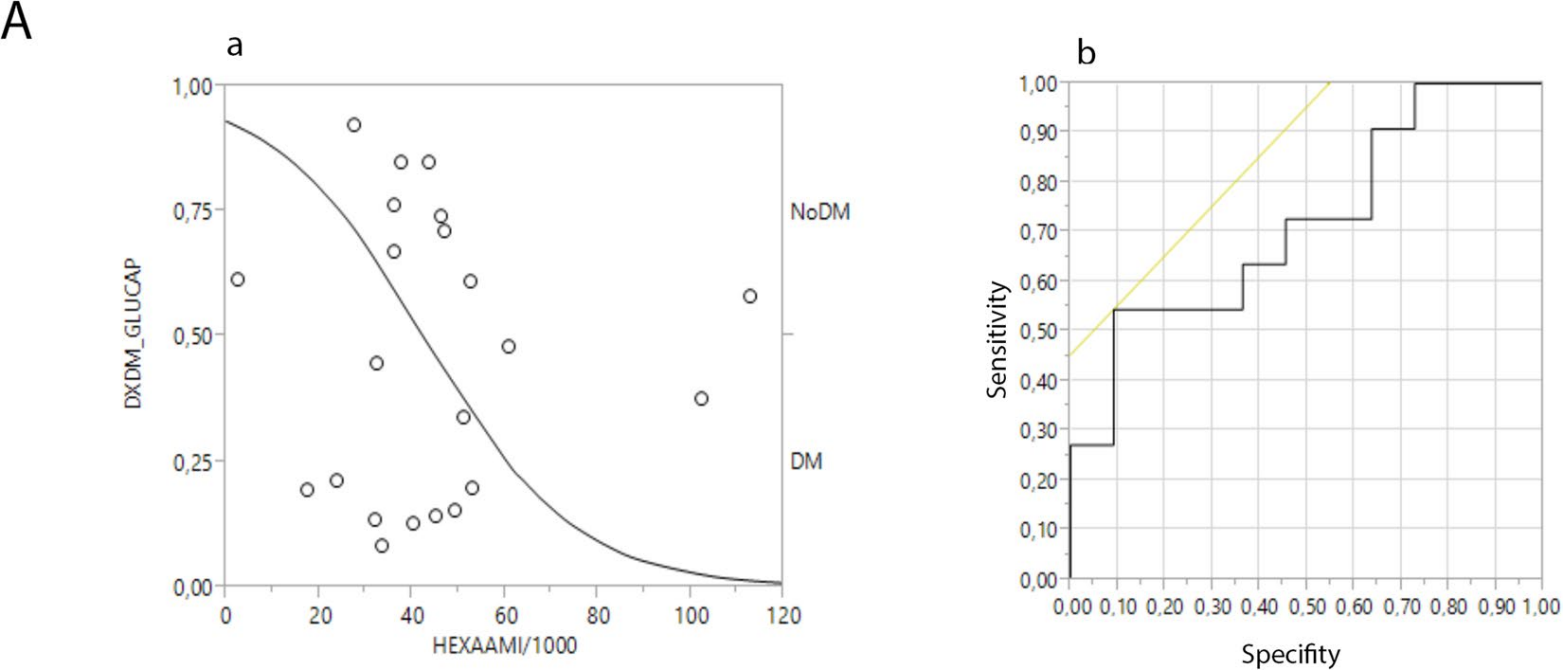
The ROC curve for the hexamers detected by the anti-IAPP antibody in serum correlated with the diagnostic bases of diabetes using capillary glucose levels. The sensitivity was 0.55 (95% CI, 0.25–0.84), and the specificity was 0.91 ([95% CI, 0.74–1.00], LR+: 6.00 [95% CI, 0.86–41.96], LR−: 0.50 [95% CI, 0.25–0.98]).

We performed receiver operating characteristic (ROC) curve analysis, which revealed that the cut-off level of 33,500 (area under the curve [AUC]: 0.72) for the hexamers detected in the serum with anti-IAPP yielded a sensitivity of 0.55 (95% confidence interval [CI], 0.25–0.84) and a specificity of 0.91 ([95% CI, 0.74–1.00], LR+: 6.00 [95% CI, 0.86–41.96], LR-: 0.50 [95% CI, 0.25–0.98], Fig. 7).

The paradigms concerning rIAPP have changed. Our results demonstrate that rIAPP undergoes anomalous folding and aggregates to form trimers and hexamers, which are predominant, although oligomers with higher molecular weights also exist (Figs 1–6). Furthermore, these aggregates are also cytotoxic oligomers that form early (i.e., after 5 hours of treatment with STZ), and their levels remain elevated during the development of diabetes (Figs 1–6). This is one of the patho-physiological bases of STZ-induced diabetes. These cytotoxic oligomers are soluble and are found in both sera and the pancreas, suggesting that they can cause cellular death in different organs (Figs 1–6).

We have thus shown by double immunofluorescence experiments, that the rIAPP it’s co-localized with the amyloid oligomers, which indicates that the rIAPP forms cytotoxic oligomers. In addition, these can be found in the nucleus as well as in the cytoplasm (Fig. 4). Interestingly, the human and rat IAPP progressive translocation in the nucleus over a period of 24 hours and the major contribution of nuclear proteasome 20S to the degradation of IAPP cytotoxic oligomers, was previously demonstrated by Singh *et al*.^46^. This suggests that proteasomes 20S could serve as well for rIAPP cytotoxic oligomer degradation in pancreatic cells of streptozotocin-induced diabetic rats. Furthermore, the interaction of IAPP amyloid oligomers with 20S proteasome could reduce proteostasis capacity, which eventually leads to proteostasis collapse^46^.

Additionally, it should be noted that rIAPP consists of homo-oligomers and hetero-oligomers (Fig. 6 and Table 1). More studies are required to understand the co-aggregation pathways^35^.

This study is one of the first to explore the rIAPP aggregation in STZ-induced diabetes in rats. We believe that the combination of methods used to explore the oligomerization of rIAPP opens a window of opportunity for the study of diabetes as a conformational disease. Furthermore, this work promotes novel avenues for the study of the physio-pathological pathways of rIAPP co-aggregation, which are currently missing from the literature but are essential to achieving an effective and deep understanding of conformational diseases. One dimension of proteomic analysis involves being attentive to other proteins that need to be identified in the co-aggregation network.

Our study opens new horizons of action in the animal model of STZ-induced diabetes from the perspective of protein folding and the patho-physiology of diabetes mellitus. Notably, using transgenic animals is not necessary to examine anomalous folding in diabetes^47, 48^ because rIAPP is aggregated in the animal model of STZ-induced diabetes.

Finally, we demonstrated that rIAPP hexamers are diagnostic biomarkers for diabetes mellitus based on capillary glucose levels (Fig. 7). Our results have implications for the diagnosis, prevention, and transmission of diabetes mellitus and for therapeutic approaches to this disease.

## Experimental Section

### STZ-induced diabetic rats

Thirty-seven female Sprague-Dawley rats weighing 150–250 g were included in the study and maintained under controlled conditions (22–25 °C, light/dark 12/12 hours and *ad libitum* access to food and water). The rats were divided into 2 groups (Experimental and Control). The experimental group (STZ) (n = 21) received an intraperitoneal (ip) injection of a single dose of 40 mg/kg STZ (Sigma-Aldrich S0130, Mexico city, Mexico) in 0.1 M acetate buffer (pH 4.3) (vehicle). Diabetic rats (>200 mg/dl) were divided in 7 groups (3 rats per group) and maintained in separate cages for 5 h, 10 h, 24 h, 36 h, 48 h, 5 days and 10 days after STZ injection. The control group (n = 16) received an ip injection of the vehicle and were divided into 7 groups (2 rats per group) and maintained in separate cages for the established times. Blood samples were obtained by cutting the tail tip, and the glucose, cholesterol, and triglyceride levels were determined before treatment. Subsequently, these levels were tested every 24 h for up to 10 days using an AccuCheck glucometer (Roche) and Accutrend Plus system (Roche).

The rats in each group were euthanised by cranio-cervical dislocation at the selected timepoints. Clinical laboratory tests (glucose, cholesterol and triglycerides) were conducted throughout the study at 0 h, 5 h, 10 h, 24 h, 36 h, 48 h, 5 days and 10 days using standard procedures in a central laboratory. All animal studies were performed in compliance with IMSS Animal Care Guideline NOM-062-ZOO 1999. The study protocol (052–55) was approved by the Ethical Committee of IMSS.

### WB

Protein extracts were prepared from the sera and pancreas of either control or STZ-induced diabetic Sprague-Dawley rats (female, 150–250 g). The pancreas was homogenized in Mammalian Protein Extraction Buffer (GE Healthcare) with a protease inhibitor cocktail (Sigma). Samples were then centrifuged at 10,000 rpm for 10 min at 4 °C. The supernatant was collected and conventional freezing in liquid nitrogen. Total protein concentration was determined by BCA assay and the concentration of the samples was normalized.

The soluble fractions of the pancreas extracts were separated by sodium dodecyl sulphate polyacrylamide gel electrophoresis (SDS-PAGE) and subsequently transferred onto polyvinylidene difluoride (PVDF) membranes. The membranes were probed overnight at 4 °C with the purified anti-amyloid oligomers antibody ab126892 (Abcam, 1:1,000) or anti-amylin antibody (Santa Cruz Biotechnology, 1:200) diluted in phosphate-buffered saline with Tween-20 (PBS-T) containing 3% bovine serum albumin (BSA). ab126892 immunoreactivity was detected with a horseradish peroxidase-conjugated anti-rabbit antibody (Pierce Poly HRP 1:20000), whereas an anti-mouse antibody (Pierce Poly HRP 1:10000) was used for anti-amylin. The membrane activity was detected by substrate chemiluminescence (Immobilion Chemiluminescence HRP Substrate 1:1) and revealed by a Li-COR C-DiGit system. The intensity of the protein bands was quantified by Image Studio Lite via scanning densitometry. The data were managed with MS Excel, while the statistics and graphs were obtained with the SAS JMP 9 statistical software package. To control the charge, we performed immunoblotting with Anti-GAPDH (Merck CB1001 1:3000) for pancreas and Anti-Transferrin (Santa Cruz Biotechnology sc-30159 1:500) for serum (Figs 1 and 2).

### Immunoprecipitation

The serum contained a protein concentration of approximately 1 µg/µL. The primary antibody (2 µL of Anti-IAPP, Santa Cruz Biotechnology) was added to the tube, and the reaction mixture was gently rocked overnight at 4 °C. The immunocomplex was captured by adding 20 µL of packed and washed Protein A agarose bead slurry. The reaction mixture was gently rocked at 4 °C for 2 hours. The sample was centrifuged at 14,000 rpm for 15 min, and then, the supernatant was drained off. The beads were washed 3 times with PBS. The agarose beads were resuspended in 50 µL of 2X SDS loading buffer and boiled for 5 minutes. The subsequent immunoblot analysis was performed on a sample of the supernatant, or the agarose beads were frozen for later use and boiled again for 5 minutes prior to SDS-PAGE.

### Proteomic analysis

The results presented here were obtained from manually cut bands. The proteins were subjected to reduction (10 mM dithiothreitol [DTT]) and alkylation (100 mM iodoacetamide) and faded with 50 mM acetonitrile (ACN):NH_4_HCO_3_ (50:50 v/v).

The protein digestion required 18 hours at 37 °C with trypsin (Promega V528A). The peptides were obtained from digestion (ACN:H_2_O:formic acid 50:45:4 v/v), and the volume of the sample was reduced in a concentrator (Eppendorf 5301). Finally, the sample was purified using a C18 column (ZipTipC18).

Each sample was spotted six times on an analysis chip using alpha-cyano-4-hydroxycinnamic acid as a matrix and analysed in a matrix-assisted laser desorption/ionization time-of-flight tandem mass spectrometer (MALDI TOF/TOF MS 4800).

Once we obtained the MS/MS spectra, we performed a search using the Paragon algorithm with ProteinPilot software based on a certain trust percentage.

### 2D SDS-PAGE

Protein (250 μg/100 μl) was precipitated with the ReadyPrep 2-D Cleanup Kit (Bio-Rad) by the addition of 300 μl of precipitating agent, thorough mixing, and incubation on ice for 15 min. Then, 300 μl of precipitating agent 2 was added to the mixture of protein and precipitating agent 1. The sample was mixed by vortexing, and the tubes were centrifuged at maximum speed (12,000 × *g*) for 5 min to form a pellet. The supernatant was removed with a pipette, and the pellet was centrifuged for 15 min to eliminate residual liquid. Subsequently, 40 μl of wash reagent 1 was added on top of the pellet and centrifuged for 5 min. The supernatant was removed, and the wash was discarded. Then, 25 μl of ReadyPrep proteomic-grade water was added on top of the pellet, and the tube was vortexed for 10–20 sec to disperse the pellet. Next, 1 ml of wash reagent 2 (pre-chilled at −20 °C) was added for at least 1 hr, followed by 5 μl of wash additive. The sample was vortexed for 1 min, and the tube was incubated at −20 °C for 30 min. During the incubation, the tube was vortexed for 30 sec every 10 min. The tube was centrifuged at 14,000 × *g* for 5 min to create a tight pellet, and the supernatant was discarded. The pellet was air dried at room temperature (RT) for no more than 5 min; pestle homogenized in 140 μl of rehydration buffer (8 M urea, 20 mM DTT, 2% 3-[(3-cholamidopropyl)dimethylammonio]-1-propanesulfonate [CHAPS], 5% Triton X-100, 0.2% ampholytes 3/10, and 0.002% bromophenol blue); and vortexed for 1 min. The tube was incubated for 1 hr at RT and centrifuged at 14,000 × *g* for 5 to 10 min at 4 °C. The supernatant was loaded through a line, and 7-cm pH 3–10 ReadyStrips (Bio-Rad) were placed gel-side down over the supernatant. Two millilitres of mineral oil were added for each line, and the sample was incubated for one hour at RT.

Isoelectrofocusing (IEF) was performed with a Protean II IEF System (Bio-Rad) programmed with passive rehydration step at 16–20 hrs at 20 °C. After the electrodes were prepared with a wet Whatman paper cover (10 μl of sterile water), the strip was taken off the supernatant, and the excess mineral oil was removed with wet Whatman paper. The strip was subsequently placed gel-side down between the electrodes, with numbers 3–10 on the cathode. Then, 2 ml of mineral oil was added, and the strip was covered with the lid. The programme was S1 Linear 250 V for 20 minutes, S2 Linear 4000 v for 2:50 hours and S3 rapid 4000 v (10,000 v/hr) for 2.5 hrs with a final rate of 10,000 Vh/hr. The total volt hours were approximately 40,000. The reduction treatment of the IEF strips was performed in 1,880 µl of equilibrium buffer (6 M urea, 0.375 M Tris HCl at pH 8.8, 2% SDS, and 20% glycerol) with 120 μl of 1 M DTT for 10 minutes at RT with gentle shaking. Alkylation treatment of the IEF strips was conducted in 1,920 μl of equilibrium buffer and 80 μl of 1 M iodoacetamide for 10 minutes at RT with shaking. The IEF strips were immediately placed in the order 3 to 10 (left to right) in front of a 15% SDS-PAGE gel in a Mini-Protean Tetra Cell chamber and run at 100 V for 2:30 hours. The gels were subjected to silver staining with the Silver Stain Plus kit (Bio-Rad) and analysed by comparing the proteins immunoprecipitated with anti-amylin from control rats and diabetic rats using the PDQuest program.

### Ultrastructural immunolocalization

Fragments of pancreas taken from control and diabetic rats were fixed with 4% paraformaldehyde in PBS for 1.5 hours at RT and subsequently washed with PBS. The samples were dehydrated with a gradual series of methanol, embedded in acrylic resin (Lowicryl K4M, EMS), and polymerized in an ultraviolet (UV) light chamber at −20 °C for 24 hours and at RT for another 24 hours. Ultrafine cuts were made, and then, the samples were placed on gold grids covered with Formvar. The grids were floated in Tris buffer solution containing 0.1% Tween-20 for 30 minutes, and immediately after this, they were floated over the primary corresponding antibody (IAPP or amyloid oligomers) in solution (1:20) overnight in a humid chamber at 4 °C. Subsequently, the grids were washed with PBS and floated over the secondary antibody conjugated to gold particles (12 nM gold particles for the antibody against amylin and 18 nM gold particles for the antibody against amyloid oligomers) (Jackson ImmunoResearch) in solution (1:100) for an hour in a humid chamber at RT. Then, the grids were washed with water by flotation and left to dry before being contrasted with uranyl acetate (3%) for 2 minutes. The grids were observed under a JEM-1010 (JEOL Japan) EM instrument, and image capture was achieved with a charge-coupled device (CCD) camera (model Gatan Orius SC600) and its digital micrograph software.

### Immunofluorescence localization of rIAPP and amyloid oligomers in pancreatic β cells

Fragments of pancreas soaked in lowicryl resin were sliced into semi fine cuts of 1 μm of thickness. The slices were made permeable with Triton X-100 (0.1%) and incubated overnight with the primary anti-IAPP antibody with a 1:100 dilution ratio. Afterwards, the slices were incubated with the secondary antibodies conjugated with ALEXA 568 for an hour with a 1:800 dilution ratio. The slices were subsequently incubated overnight with the primary anti-amyloid oligomers’ antibody (1:100) and later incubated with the secondary antibody conjugated with FITC (Dako Corporation) (1:200) for one hour. To finish up 4′,6-diamidino-2-phenylindole (DAPI) (0, 1 μg/ml for two minutes) was added and mounted with vectashield (Vector Laboratories, Inc.). The images were acquired using a confocal Leica TCS SP8 microscope using a 63X/1.40 oil immersion objective lens and LAS × 2.0.2.14392 software.

### Amyloid fibril histochemistry with Congo red

Semi-fine 2-μm-thick cuts were obtained, mounted on a slide, and fixed with heat. The samples were then dyed with a few drops of 0.5% Congo red solution for 30 seconds while being heated. Then, the samples were washed with deionized water, counterdyed with Gill haematoxylin for another 30 seconds, and washed again with deionized water. The cuts were dried with heat and mounted with synthetic resin. Image capture was achieved with an Axiostar microscope (Carl Zeiss) equipped with a Canon EOS-1000D camera and its corresponding program.

### Data availability statement

All relevant data are within the paper and its Supporting Information file.

### Ethical approval

All animal studies were performed in compliance with IMSS Animal Care Guideline NOM-062-ZOO 1999. The study protocol (2010-785-052 was approved by the Ethical Committee of IMSS.

## Electronic supplementary material

Supplementary Information
