# Supplementary material for: Novel insight into streptozotocin-induced diabetic rats from the protein misfolding perspective
Source: Sci Rep. 2017 Sep 14;7:11552. doi: 10.1038/s41598-017-11776-y (PMC5599686; doi:10.1038/s41598-017-11776-y)
Supplement: Supplementary file 1 — Supplementary Information [file 41598_2017_11776_MOESM1_ESM.pdf]

Novel insight into streptozotocin-induced diabetic rats from the protein misfolding perspective

Edgar Leyva-García[#] Reyna Lara-Martínez[&], Liborio Morán-Zanabria[#], Cristina Revilla-Monsalve[#], Luis Felipe Jiménez-García[&], Norma Oviedo[^], Chiharu Murata[%], Eulalia Garrido-Magaña[|], Nelly F. Altamirano-Bustamante[%,*], Myriam M. Altamirano-Bustamante[#,*].

[#]Unidad de Investigación en Enfermedades Metabólicas, Centro Médico Nacional Siglo XXI, Instituto Mexicano del Seguro Social, Mexico city, Mexico

[&]Facultad de Ciencia – UNAM, México city, Mexico

[^]Unidad de Investigación en Inmunología e Infectología. Centro Médico Nacional La Raza, Instituto Mexicano del Seguro Social, Mexico city, Mexico

[%]Instituto Nacional de Pediatría, México city, México

[|]Servicio de Endocrinología, UMAE Hospital de Pediatría, Centro Médico Nacional Siglo XXI, IMSS. Mexico city, Mexico;

[*]Corresponding authors

Figure Legends

Supplemental Figure 1. Shows the changes of level of glucose in STZ-induced diabetes rat and control rats during 10 day

Supplemental Figure 2. Shows a comparison of the curves relating to the body weight in two groups of control and STZ-treated rats.

Supplementary Figure 1

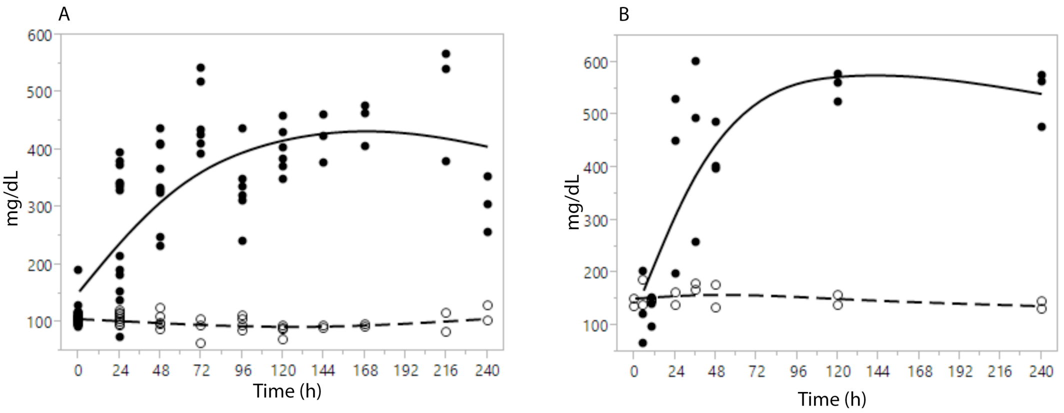

Supplementary Figure 2

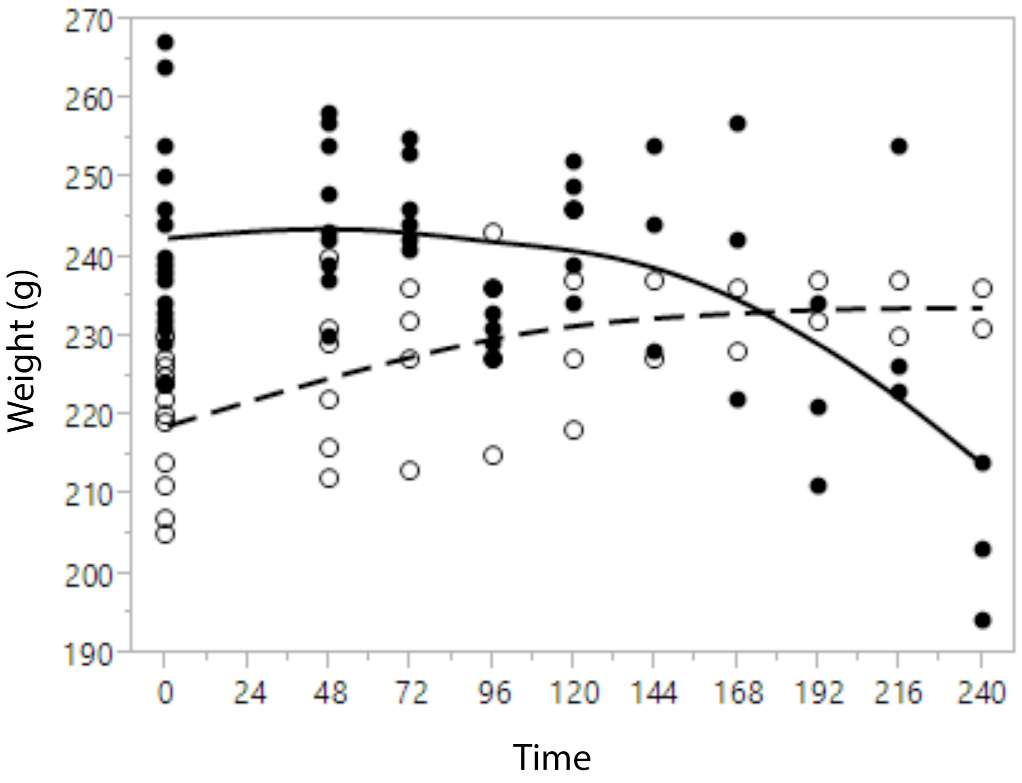

Weight (g)
270
260
250
240
230
220
210
200
190
0
24
48
72
96
120
144
168
192
216
240
Time
